# Assessing the Depth of Cognitive Processing as the Basis for Potential User-State Adaptation

**DOI:** 10.3389/fnins.2017.00548

**Published:** 2017-10-04

**Authors:** Irina-Emilia Nicolae, Laura Acqualagna, Benjamin Blankertz

**Affiliations:** ^1^Department of Applied Electronics and Information Engineering, Politehnica University of Bucharest, Bucharest, Romania; ^2^Department of Neurotechnology, Technische Universität Berlin, Berlin, Germany

**Keywords:** encephalography (EEG), event-related potentials (ERPs), oscillatory activity, cognitive processes, single-trial classification

## Abstract

**Objective:** Decoding neurocognitive processes on a single-trial basis with Brain-Computer Interface (BCI) techniques can reveal the user's internal interpretation of the current situation. Such information can potentially be exploited to make devices and interfaces more user aware. In this line of research, we took a further step by studying neural correlates of different levels of cognitive processes and developing a method that allows to quantify how deeply presented information is processed in the brain.

**Methods/Approach:** Seventeen participants took part in an EEG study in which we evaluated different levels of cognitive processing (no processing, shallow, and deep processing) within three distinct domains (memory, language, and visual imagination). Our investigations showed gradual differences in the amplitudes of event-related potentials (ERPs) and in the extend and duration of event-related desynchronization (ERD) which both correlate with task difficulty. We performed multi-modal classification to map the measured correlates of neurocognitive processing to the corresponding level of processing.

**Results:** Successful classification of the neural components was achieved, which reflects the level of cognitive processing performed by the participants. The results show performances above chance level for each participant and a mean performance of 70–90% for all conditions and classification pairs.

**Significance:** The successful estimation of the level of cognition on a single-trial basis supports the feasibility of user-state adaptation based on ongoing neural activity. There is a variety of potential use cases such as: a user-friendly adaptive design of an interface or the development of assistance systems in safety critical workplaces.

## Introduction

While Brain-Computer Interface (BCI) research primarily targets medical applications (Birbaumer, 2006; Dornhege et al., 2007; van Gerven et al., 2009; Wolpaw and Wolpaw, 2012; Guger et al., 2014; Hassanien and Taher Azar, 2015), more and more perspectives are being explored that go beyond communication and control paradigms (Blankertz et al., 2010, 2015, 2016; Zander and Kothe, 2011; Borghini et al., 2014; Zander et al., 2016). One of those perspectives are systems that take the ongoing user mental state into account and automatically adapt according to the user's mindset (Müller et al., 2008; van Erp et al., 2015) exploiting implicit information (Gamberini et al., 2015).

Neural correlates of cognitive processes can be found in the time-locked Event-Related Potentials (ERPs), usually composed of different components, and in modulations of spontaneous brain rhythms. The phase of those background rhythms is not time-locked to external stimuli, but the modulation of their amplitudes (resp. their hull curves) can be time-locked. In that case, the effect is called Event-Related Desynchronization or Synchronization (ERD/ERS) (Pfurtscheller and Lopes da Silva, 1999; Lemm et al., 2009), depending on whether it is a decrease or an increase in spectral power of the given frequency band.

ERP components have been widely investigated and previous studies involving cognitive activities in oddball paradigms show that the amplitude and latency of the ERPs are modified according to task difficulty (Donchin et al., 1973; Ullsperger et al., 1987; Polich, 2007; Kim et al., 2008). Specifically, increased P300 amplitude and longer latencies are usually found, relating to more complex processes and stronger attentional demand. These cognitive ERP components are reported to appear between 300–500 ms after the stimuli, in the centro-parietal cortex (Polich, 2007).

Moreover, changes in frequency are also reported, being modulated by the difficulty of cognitive processes. Firstly, the presentation of a stimulus triggers a short synchronization proceeded by a prolonged desynchronization mostly occurring in the α band at the frontal, temporal, central, and parietal locations of the scalp in accordance with the type of cognitive process (Klimesch et al., 1992, 1993, 1997; Klimesch, 1999), and follows the P300 potential (Yordanova et al., 2001). The α band desynchronizes during mental activity and cognitive judgment (Klimesch, 1999), and is reported proportionally increasing with more difficult cognitive processing, change mostly encountered at the centro-parietal sites. The effect is visible in laboratory environments (Gevins et al., 1997) and as well in more realistic scenarios (Venthur et al., 2010).

In addition, β oscillations are also linked to complex cognitive processes (Pesonen et al., 2007; Okazaki et al., 2008; Sheth et al., 2009), whereas decreased β oscillations appearing at the central and parietal sites are correlated to complex reasoning (Basile et al., 2013), decision making (Nakata et al., 2013) and are also related to the transition of cognitive states (Sheth et al., 2009).

Changes in the θ activity are observed as synchronizations in relation to task difficulty (Klimesch, 1999), e.g., increased θ power proportionally with increased memory load (Gundel and Wilson, 1992; Gevins et al., 1997), which also relate to the encoding of new information (Klimesch et al., 1996; Klimesch, 1999). Regarding localization, the θ changes are known to appear at the frontal midline scalp location (Gevins et al., 1997).

A pronounced ERD was found in relation to different types of cognitive processes. For example, in memory processes (Mecklinger et al., 1992; Klimesch et al., 1994; Stipacek et al., 2003; Pesonen et al., 2007), perceptual encoding and attentional processes, a stronger ERD is observed in the α band (Sergeant et al., 1987; Klimesch, 2003; Schack et al., 2005; Polich, 2007). In addition, the processing of semantic information, e.g., words, shows likewise ERD enhancements (Klimesch et al., 1997).

In a recent study, Naumann et al. (2017), investigated gradual differences in task difficulty by estimating the difficulty level of a video game from the ongoing neural activity of the user. They found likewise significant modulations in the θ (4–7 Hz) and α (8–13 Hz) frequency bands, associated with changes in task difficulty.

In the present work, we have studied the feasibility of quantifying how deeply presented information is processed in the brain by tapping the corresponding components of brain activity. To that end, we analyzed single-trial EEG data with respect to its discriminative value of ERPs and of modulations of brain rhythms. In the ERPs, we found as main effect an increase of the P300 amplitude with task demand and also domain specific modulations in later components, see also (Nicolae et al., 2015a). Spontaneous background oscillations showed a prolonged suppression of the α and β rhythm as a reflection of profound cognitive processing. For extracting the features for classification, we employed spatio-temporal features of the ERPs (Blankertz et al., 2011) and we exploited the ERD effect by combining spatio-spectral decomposition (SSD, Nikulin et al., 2011) in two frequency bands with Common Spatial Patterns (CSP) analysis (Fukunaga, 1990; Koles, 1991). Combining features of ERPs and ERDs in a multimodal classification approach (Fazli et al., 2015) leads to a performance increase compared to using a single modality only (Nicolae et al., 2015b). The current manuscript comprises a detailed investigation of the depth of cognitive processing integrating the analysis over the modulations of brain rhythms (ERDs) and the spectral analysis discriminability (power spectrum, CSPs), extending previous work in Nicolae et al. (2015a), where the temporal and spatial evolution of the ERPs are briefly analyzed and the abstract in Nicolae et al. (2016), where a short overview over the power spectrum is presented. In addition, the present work contributes further with an enhanced multivariate classification approach based on both, the temporal and spectral features extracted from the EEG, as compared to the classification based on only the temporal features presented in (Nicolae et al., 2015b).

## Materials and methods

### Experimental setup

#### Participants

Fifteen healthy participants with no acute or chronic neurological and/or psychiatric disorders and no pregnant women were considered for the study. Eleven participants were right-handed, ten were males and all were aged between 22 and 35 years old. Eleven participants had German as mother tongue, one participant had English and the others had different languages as native tongue, with a required good command of English or German in order to fulfill the task in the language condition. Seventeen participants were initially recorded, but two participant's data were removed for the analysis due to high artifacts probably caused by improper recordings. The experimental procedure was conducted in accordance with the declaration of Helsinki, approved by the ethics committee of the Department of Psychology and Ergonomics of the Technische Universität Berlin and written informed consent was obtained from each participant. To countenance participant's motivation, they received financial compensation.

#### Material

The hardware equipment used for the acquisition of the Electroencephalography (EEG) was a BrainAmp amplifier with 64 active electrodes (Brain Products GmbH, Munich, Germany) positioned according to the 10–20 international system. One electrode, named *EOG*, was placed under the left eye and used for eye movements recording. We used unipolar recording at a sampling frequency of 1 kHz with the ground placed on the scalp at position AFz and with reference at left mastoid. The acquisition system was re-referenced to left and right mastoids. When mounting the electrode cap, the impedance was kept below 20 kΩ. The acquired data will be made available from the DepositOnce repository of Technische Universität Berlin (https://depositonce.tu-berlin.de/).

The stimuli were designed as vectorial graphics with the *Inkscape* software (version 0.91.0.1 https://inkscape.org) besides the images from the animals category which were created by *Freepik* and taken from the *Freepik* database (http://www.freepik.com/free-photos-vectors/icon). The stimuli were presented on a 24” display with 60 Hz refresh rate and 1,920 × 1,200 resolution (Dell U2410). For developing the experimental paradigm, the *Processing* software (version 3.0a4 https://processing.org/) was used in conjunction with MATLAB software for signal acquisition (release R2014a, The MathWorks, Inc., Natick, MA, USA). For the offline analysis, the data was processed with the BBCI MATLAB Toolbox (https://github.com/bbci/bbci_public).

#### Experimental scenario

**Figure 1:**
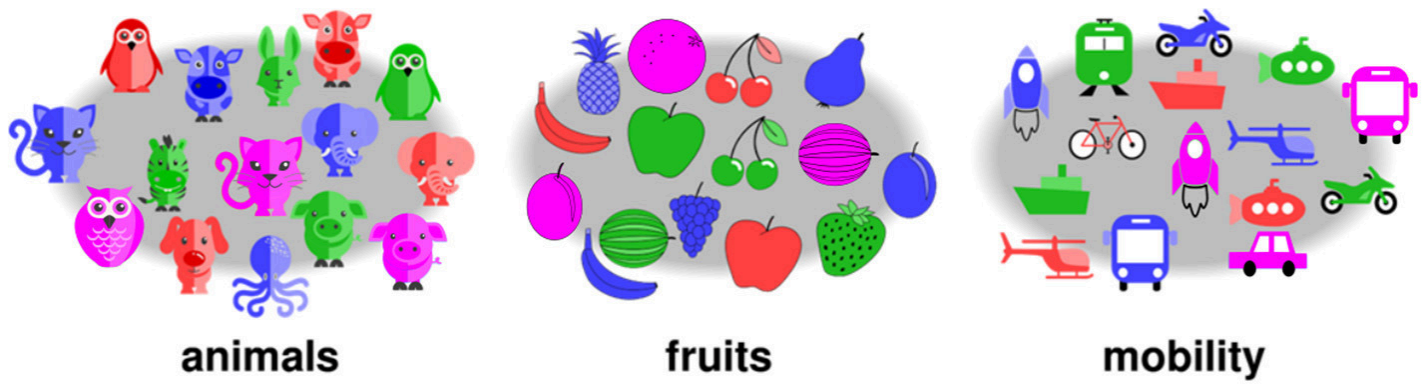
Stimuli categories (animals, fruits, mobility) and objects representations examples. Each object could be presented in one of the four colors (red/green/blue/magenta).

We considered two degrees of cognitive processing, namely shallow and deep processing levels (Craik and Lockhart, 1972) in a visual stimuli paradigm. In our scenario, the shallow processing involves a basic information processing revealed by attention (color appearance) and deep processing requires a complex activity related to specific cognitive tasks in the *memory, language*, and *visual imagination* conditions (Ganis et al., 2013). Each visual stimulus consisted of a pair of two images, cartoon-like drawings and every image represented an object chosen out of three categories: animals, fruits and mobility and was represented in one of the colors: red, green, blue, or magenta. Both images had same color and same category, where each category consisted of a total of 10 objects, see Figure 1. In order to maintain the desired ratio and without increasing too much the difficulty of the tasks, only two out of the three categories were chosen for each run.

The experimental study took place in a laboratory environment. Participants performed the experiments seated, and were requested to stay still, relaxed, and focused. They were allowed to freely explore the information presented, but in general, to focus their view in the center of the screen. Each participant was preliminary prepared with a practice test (1–3 runs) in order to become familiar with the tasks.

**Figure 2:**
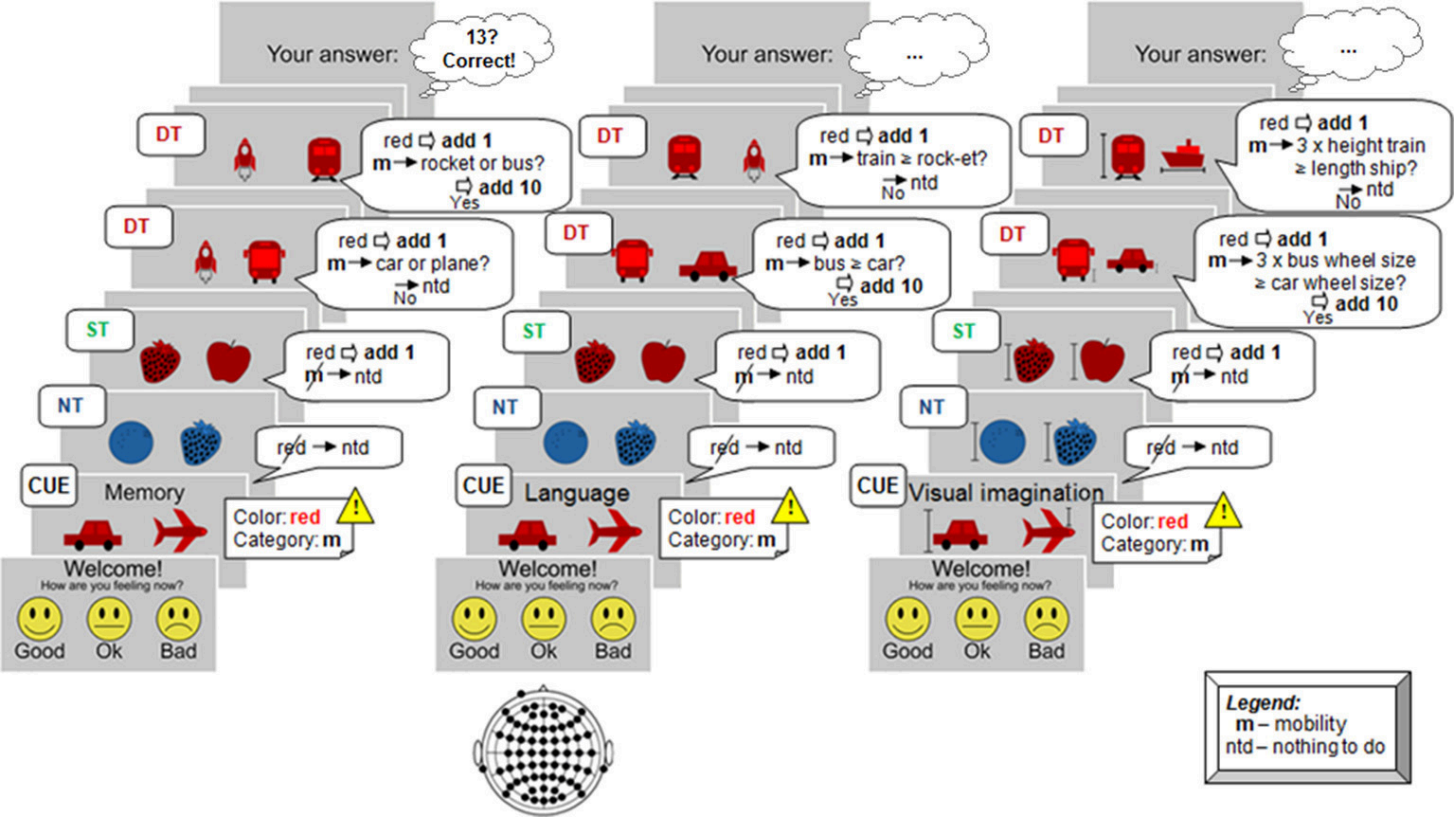
Example of experimental protocol. At each stimulus participants had to perform or not a specific task, involving mental computation as a result. The total number counted was inserted at the end of the run. From left to right, the cognitive processes are illustrated here: *memory* case (recall *memory* and compare with the last target pair), *language* (compare the number of syllables), and *visual imagination* (imagine the objects in reality and perform dimensions comparison).

The experiment paradigm is described below. Before the start of the sequence, and after a short personal current state evaluation, the condition to be performed was displayed along with the target image pair (target color and category). When the sequence started, participants had to distinguish first between color, and subsequently for category. If the color does not match the target, then no processing at all was requested (**Non-Target, NT** case). If only the color matched, perform only mental computation (**Shallow Target, ST** case). If color and category matched the target (**Deep Target, DT** case), then evaluate the requested cognitive task and perform the corresponding mental computations (addition). Following the example sketched in Figure 2, the procedure was as follows:

1. Target pair (cue): remember the target category, target color and the target images;
2. For each stimulus:
  ◦ First, check if the stimulus color matches the target *color*:
    → if not, do nothing (**NT**—non-target);
    → if yes, count +1 and:
      ▪ check if the category matches the target *category*:
        → if not, do nothing (**ST**—shallow target);
        → if yes, perform the *cognitive task* associated to each cognitive process and memorize the new images for the next trial in case of the *memory condition*:
          - if the answer is negative, do nothing additional (**DT**—deep target);
          - or in case of positive answer, additionally count +10 (**DT**—deep target);

The resulting final number was inserted at the end of each run, followed by the feedback regarding the correct number.

The cognitive *conditions* were separately conducted in this order: *memory, language*, and *visual imagination*, with five runs per condition. We did not alternate between the conditions after each run in order to avoid confusions between the tasks, which have been quite demanding. In each *condition* participants had to “answer” a yes-no question, which is detailed hereunder.

##### Memory

The first *condition* considers memory retrieval by comparing a previously presented stimulus with the current stimulus (Kirchner, 1958; Chen et al., 2008). Specifically, the question is whether the current stimulus coincides with previous *target pair* (last pair of *target color* and *target category*). The accomplishment implies memorization of the current pair and retrieval of the previous target part. For an example see Figure 2, left column. An additional example of the *memory* experimental paradigm is provided in Supplementary Video 1 of the Supplementary Presentation 1.

##### Language

The *language* task considered comparisons based on phonemic representations between the words that represent the images. The task was to decide whether the number of syllables of the left image's word was greater or equal than the number of syllables of the word for the object on the right side. This *condition* considered English or German words based on the participant's native *language*. Examples are shown in Figure 2, middle column. Note that the chosen objects (Figure 1) have a quite unique mapping to their representing words. In case a test person employed a different word, that could just affect the behavioral data, while the actual performance (DT vs. ST or NT) is not affected. Additional example for the *language* experimental paradigm is encountered in Supplementary Video 2 of the Supplementary Presentation 1.

##### Visual imagination

The *visual imagination* task required mental representations in order to perform a comparison based on the size in reality. The differentiations made are accomplished by judging whether three times the dimension of the left object (or a part of the object) is greater or equal to the right object's size, considering average dimensions of the represented objects. The respective part of the object and the dimension type (length, height, or thickness) used for comparison, was emphasized with a marker on the stimulus image. Further examples are in Figure 2, right column.

When generating the stimulus pairs for the *visual imagination condition*, a small constraint was added in order to ensure similar complexity within the *visual imagination* task. This related to the differences of dimensions within a range, more exactly the absolute difference between three times the left object and the right object should be less or equal than the left object dimension. Hence, no big discrepancy between the objects sizes was assured: no large differences implying an easy comparison, and no small ones either, which would result in a hard or ambiguous comparison. Short additional example for the *visual imagination* experimental paradigm is encountered in Supplementary Video 3 of the Supplementary Presentation 1.

#### Experimental design

**Figure 3:**
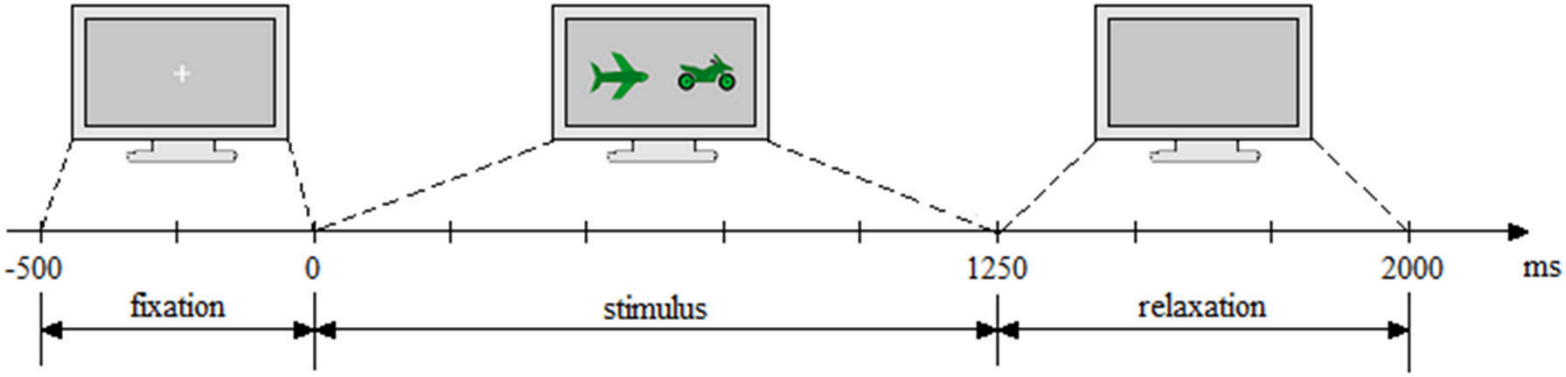
Experiment timing. First, the fixation cross was displayed (500 ms), followed by the trial execution (1,250 ms) and ended with the relaxation period (750 ms, light gray blank screen).

As a pilot study, four participants were asked to test the application without EEG cap. The results of this pilot study have been used to calibrate the speed and the complexity of the task. The time course of the experiment is presented in Figure 3. It consisted of 2,500 ms Inter-Stimuli Interval and was divided as follows: 500 ms fixation, 1,250 ms stimulus presentation, and 750 ms relaxation period.

Each condition was evaluated in five runs with a total of 600 stimuli. The two images representing the stimulus, were scaled to a common 480 × 480 resolution and presented close to the center of the screen at a distance of 2” from one another on a light gray background.

The same percentage of shallow and deep targets was chosen in order to avoid confounds caused by a different number of occurrences: 75 ± 2% for non-targets (NT) and 12.5 ± 2% for both, shallow targets (ST) and deep targets (DT).

### Behavioral assessment

The subjects behavioral responses were assessed by subjective and objective indicators. The subjective indicators that we took into account by means of a questionnaire are: personal feedback, meaning personal internal state/mood (good/ok/bad) and personal overview for the difficulty of the conditions, which was acquired by scoring (0—easy; 1—medium; 2—hard). The objective indicator that we integrated (subjects answers ratio), is given by the actual responses, assessed by a ratio of the absolute difference between the correct number and user response number, divided by the correct number.

### Data analysis

Hereunder, the signal processing and machine learning methods are described, mainly the multi-modal analysis and multivariate classification which give us complementary and additional information over the neurophysiological effects of the cognitive processes. The multi-modal analysis investigates the neural correlates from the temporal and spectral domain: ERPs and Event-Related (De)Synchronization, ERDs. The brain sources of neural oscillations which substantiate cognitive activity (Varela et al., 2001; Buzsáki and Draguhn, 2004) were extracted by advanced decomposition methods: SSD by Nikulin et al. (2011) and Common Spatial Pattern (CSP) (Fukunaga, 1990). The two different types of neurophysiological information, temporal, and oscillatory activity, are combined with the concatenation approach as described in Dornhege et al. (2004), in order to give better performance as shown for example in other studies (Mühl et al., 2014). Finally, the depth of cognitive processing of the external information is estimated using multivariate single-trial classification, by Regularized Linear Discriminant Analysis (Friedman, 1989).

#### Filtering and epochs rejection

For dimensionality reduction, the data was downsampled to 100 Hz. Preliminary processing of the data was performed by a sequence of low-pass filtering for anti-aliasing and high-pass filtering for drifts removal. The low-pass filter design for 42 Hz, is a Chebyshev type II of order 10 with 42 Hz pass-band edge frequency and 3 dB ripple, and a 49 Hz stopband with 50 dB attenuation. For high-pass filtering, a 1 Hz FIR filter of order 300 (three times the downsampling frequency) was applied, using least-squares error minimization and reverse digital filtering with zero-phase effect (For future online classification, appropriate causal filters must be considered). Following, the data was segmented into epochs considering the experiment timing detailed in Figure 3.

A rough pre-cleaning of the data was additionally performed obviating noisy channels and epochs. A criterion evaluated on band-pass filtered data in a broad frequency band (5–40 Hz) was applied to remove the channels dropping to zero. Particularly, the channels with variance smaller than 0.5 μV^2^ in more than 10% of the trials were removed. Moreover, the epochs with muscle artifacts were also removed considering the trials with excessive variance in 20% of the channels. For strong eye movements artifacts, max-min criterion was applied and epochs with more than 150 μV difference between maximum and minimum voltage in channels F9, F10, AF3, and AF4 were removed. For removing the background noise of the remaining epochs, baseline correction was performed trial-wise by subtracting the mean amplitude computed on 100 ms of the pre-stimulus trial period from each trial period time point.

#### Artifact removal

As a next step for removing the artifact data, including smaller eye movement artifacts, muscular artifacts and loose electrodes we performed Independent Component Analysis (ICA) with artifactual components selection given by the Multiple Artifact Rejection Algorithm (MARA, Winkler et al., 2011). For further verification, visual inspection was performed over each component considering the power spectral density and its topographic distribution.

#### Univariate discriminative analysis

In addition to the temporal ERP analysis in Nicolae et al. (2015a), we evaluated the differences among levels of cognitive processing in the spectral domain (Nicolae et al., 2016). Moreover, we investigated the power spectrum with a discriminative measure given by the signed and squared point biserial correlation coefficient (signed *r*^2^) which quantifies the discriminability between the two classes and the time course of power modulations with the help of Event-Related De/Synchronization (ERD/ERS) curves (Pfurtscheller and Lopes da Silva, 1999) in selected frequency bands.

#### Multivariate classification and validation

In order to join complementary information about the neural activity and therefore improve single-trial classification, we combined two different types of neurophysiological information. We considered spatio-temporal features reflecting ERPs and oscillatory activity features, which are described hereunder.

##### Spatio-temporal features

The spatio-temporal features (channels and time) were extracted as in Nicolae et al. (2015a). The method, further described in Blankertz et al. (2011), detects five temporal windows for each participant based on a heuristic selection of the intervals with maximum discriminability and a constant pattern between two classes based on the signed and squared point biserial correlation coefficient (signed *r*^2^). The selected intervals contain the most significant spatio-temporal features, effective as found in other studies (e.g., Acqualagna and Blankertz, 2013).

##### Spatio-spectral features

As the cognitive processes produce observable modulations in the oscillatory activity, we considered extracting this information from the respective frequency bands. Based on the discriminative analysis, we selected the most significant frequency bands: α (8–14 Hz) and β (16–20 Hz). To enhance the discrimination of activity in the frequency band of interest, we performed SSD (Nikulin et al., 2011) and Common Spatial Pattern (CSP) (Blankertz et al., 2008) for the corresponding frequency bands.

*Spatio-spectral decomposition, SSD*. Linear spatial filtering prior to CSP was used in order to reach an efficient differentiation of mental states depicted by ERD/ERS rhythm patterns. Neural activity can be sometimes concealed in the background noise fluctuations, and therefore, for a better discrimination of the neural oscillations regarding cognitive processing, we applied SSD (Nikulin et al., 2011) which enhances the signal-to-noise ratio. In order to extract individual oscillatory sources, SSD finds the optimal spatial filters based on a generalized eigenvalue decomposition, that relates to high band power in the frequency of interest (pass-band filter) and low band power in the noise in the adjacent frequencies. The adjacent noise frequencies reduction is obtained with two pass band filters of a desired width (e.g., 1 or 2 Hz) below and, respectively, above the frequency of interest, before or after a gap (stop-band filter of e.g., 1 Hz) just below, respectively, just above the frequency of interest. Important notice: because SSD requires frequency filtering in advance, we used continuous data to avoid filter edges artifacts.

For the SSD decomposition, we consider only components with eigenvalues higher than 10^−6^ times the highest eigenvalue (see low-rank factorization in Haufe et al., 2014). Typically, between 15 and 35 components per discrimination pair were further selected.

*Multi-band common spatial patterns, mcsp*. In order to obtain discriminative information about the cognitive processes based on oscillatory activity, we make use of the widely used CSP method as described by Fukunaga (1990) and Koles (1991), which was successively applied in a similar context (Schultze-Kraft et al., 2016). CSP facilitates the binary discrimination of different brain states by spatial filtering, enhancing the signal of interest while suppressing the background activity, by maximizing the variance for one class whilst minimizing the variance for the other class and vice versa. In our case, it increases the variance of a higher level of cognitive processing while diminishes the variance of a lower level of processing and vice versa. The components reaching this goal were automatically selected (as in Blankertz et al., 2008) up to a maximum of three spatial filters per class.

Based on the two relevant frequency bands, α (8–14 Hz) and β (16–20 Hz), we extracted the most discriminative spatial filters within each band and we combined them, such that the neural components referring to both frequency bands could be simultaneously exploited. The entire process was performed on the data after applying band-pass and SSD filters. The time interval considered was selected from the ERD/ERS phenomena, starting from 350 ms after the stimuli, which corresponds roughly to the peak time point of the P300, from which point the cognitive process should generally begin (Nicolae et al., 2015a).

##### Combined spatio-temporal and spatio-spectral features and evaluation scheme

**Figure 4:**
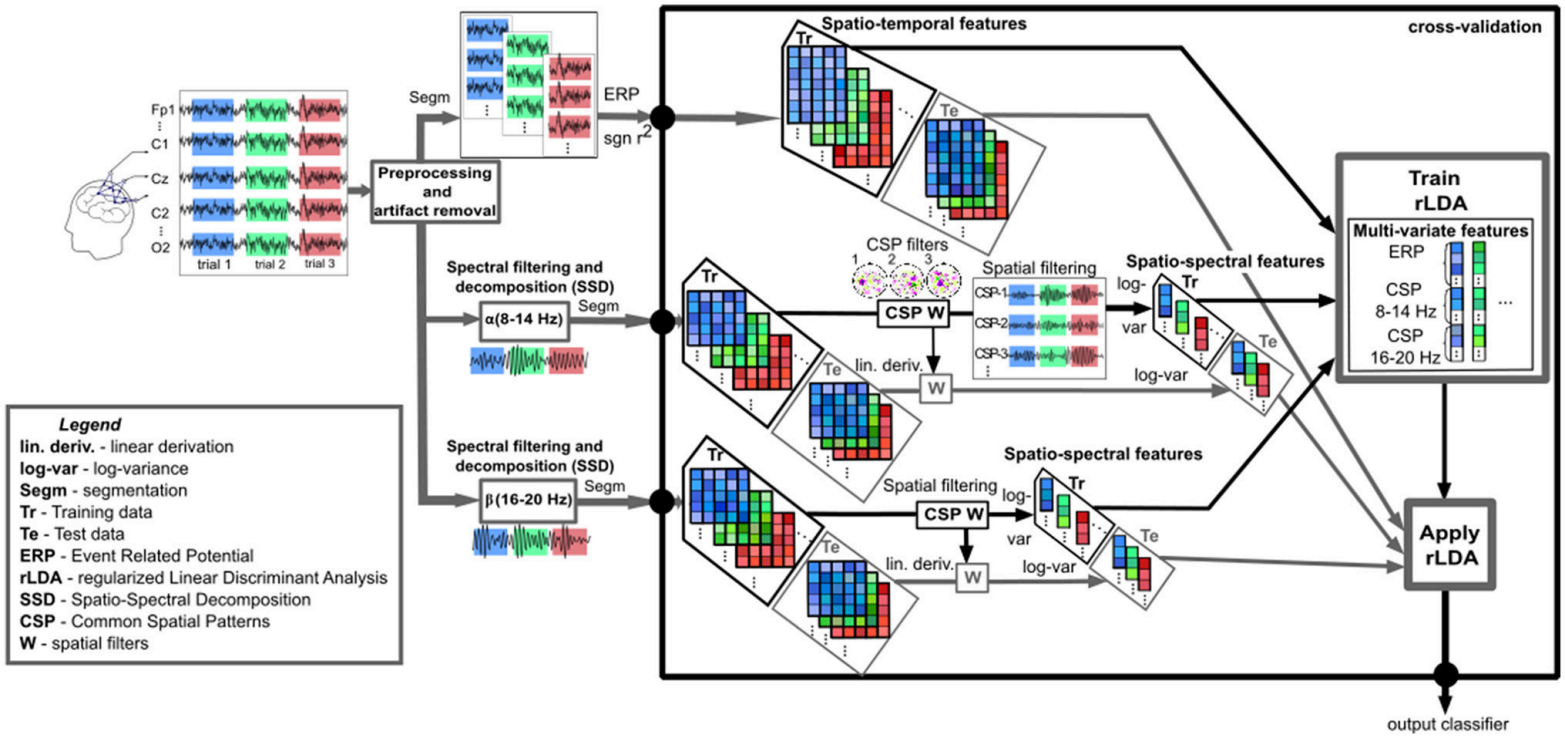
Outline of the data analysis chain. The preprocessed neural data is analyzed in the temporal domain (ERPs based on signed *r*^2^) and the spectral domain (spectral filtering and decomposition in the α (8–14 Hz) and β (16–20 Hz) bands). All three types of feature vectors: the spatio-temporal features (meaning the most important temporal points based on the maximum signed *r*^2^ intervals, the spatio-spectral features (log-variance of the CSPs) in the α and β bands, are concatenated and given to the classifier within crossvalidation. Due to label information employment, spatio-spectral filtering considers the optimal channel and frequency band using CSP analysis with automatic filter selection computed on the training set (CSP W) and applied to the test set by linear derivation (W). SSD and the interval selection method based on signed *r*^2^ were applied to the whole dataset and not within crossvalidation. While this aspect of the validation is not perfectly sound, the expected overestimation of the performance is limited. Finally, the classifier (regularized Linear Discriminant Analysis with shrinkage of the covariance matrix) decides the corresponding class membership for each trial (output classifier), representing the cognitive processing level.

Targeting the estimation of user's cognitive processing, we followed the processing pipeline described in Figure 4. After appropriate preprocessing for each feature type (time or power), the spatio-temporal features were combined with the spatio-spectral features given by the mCSP process, which were then classified and evaluated by cross-validation. More specifically, considering the band-power domain, the relevant spectral (log-variance) and spatial features were detected from the training data and used to spatially filter the testing data with the corresponding CSPs and were applied repeatedly in cross-validation manner (10 folds with 10 repetitions). Further, the separation was performed by a regularized Linear Discriminant Analysis (Friedman, 1989; Lemm et al., 2011) with shrinkage of the covariance matrix (Ledoit and Wolf, 2004; Schäfer and Strimmer, 2005; Vidaurre et al., 2009; Blankertz et al., 2011), which proved to be successful for this type of analysis (Müller et al., 2003; Bartz and Müller, 2013; Farquhar and Hill, 2013). The classification performance, as the amount of correct estimated trials, was measured by the area under the Receiver Operating Characteristic (ROC) curve (Hanley and McNeil, 1982).

Initially, no normalization was performed on the feature vectors, because in our case, the features are roughly on the same scale. However, z-score normalization of the feature vectors was also applied as comparison, by subtracting the mean and dividing by the standard deviation on each feature type.

## Results

### Behavioral data

**Figure 5:**
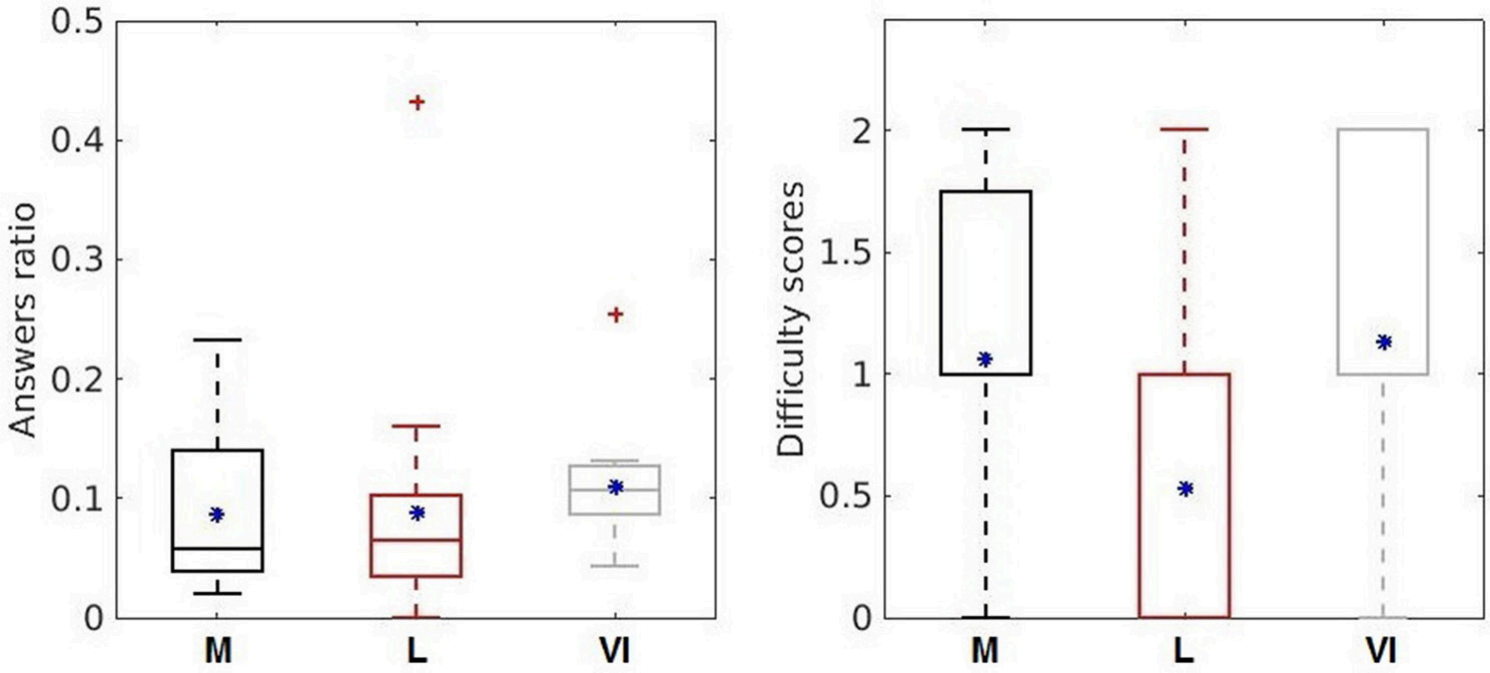
Behavioral assessment indicators: answers ratio **(left)** and difficulty scores **(right)** for the three cognitive processes (memory—dark gray, language—light red, visual imagination–light gray). The upper and bottom whiskers of each box-plot corresponds to the maximum and minimum values over all participants. The horizontal sides of the rectangular boxes represent the 25 and 75% percentiles of the data. The mean values are represented by the blue asterisk (^*^) and the outliers are indicated by the red crosses. Left plot of answers ratio is taken from Nicolae et al. (2015b) with permission from Springer.

Regarding the subjective indicators about the difficulty of the conditions (Figure 5, right), the lowest score was attributed to the *language* condition, the 25% percentile shows 0 score difficulty and the 75% shows a medium difficulty with score 1, meaning that the subjects mostly considered *language* as the easiest condition. The *memory* and *visual imagination conditions* were considered equally difficult by the subjects (25% rated a medium difficulty with score 1 and around 75% rated a high difficulty with score 2). For the personal user mood evaluation, it was reported a good mood in 53% of all the experiment runs and subjects, and in 47% it was specified as “ok.” No bad mood was reported by the subjects during or after the experiment.

Considering the objective method given by answers ratio, we observe more accurate answers for the *language* condition with a ratio closer to zero (Figure 5, left—the answers ratio for all participants averaged over the runs for each condition). No improvement or decrease in performance was observed over time considering the answers ratio, showing insignificant correlations by the Spearman rank-order correlation (*memory*: *p* = 0.3367; *language p* = 0.3982; *visual imagination p* = 0.1211; and in total over all 15 runs: *p* = 0.6192). However, most of the participants showed engagement and enthusiasm throughout the experiment.

### Neurophysiological data

**Figure 6:**
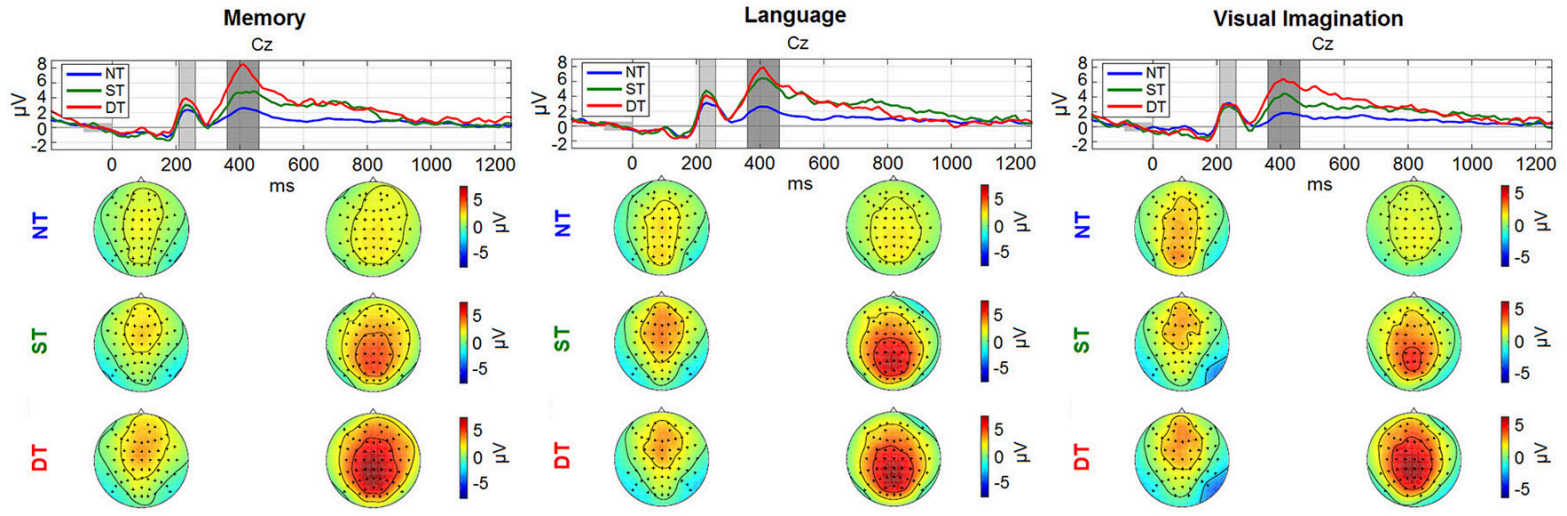
Grand average ERPs: (a) *Memory condition*; (b) *Language*; (c) *Visual imagination*. The upper plots represent the time evolution at representative electrode Cz for the non-processing (blue line), shallow processing (green), deep processing (red). The scalp plots underneath show the topographies (−2 to 8 μV^2^) referring to the shaded areas (210–260 ms; 360–460 ms) highlighted in the time plots. The figure (with scaling adjustments) was taken from Nicolae et al. (2015a), with permission from the IEEE Proceedings.

The temporal and spatial distribution of the neural activity represented by the ERPs is shown in Figure 6 (with more details in Nicolae et al., 2015a).

Looking at the spatial and temporal distribution of the ERPs in Figure 6, a gradual difference between the levels of cognitive processing is observed in the centro-parietal area reflecting a positive peak about 400 ms after the stimulus (~P300). Earlier, a peak around 250 ms is observed, with a negative component more pronounced in the right-occipital cortex, discriminating between no processing and processing, and similarly in the *visual imagination condition*, but discriminating also between shallow and deep processing.

Second, we visualized the modulations of the signals' power spectrum computed for the entire trial timing (2 s) in a spatial-spectral representation (topographic maps). In order to focus on the discriminative aspects, the visualization was based on signed *r*^2^-values.

**Figure 7:**
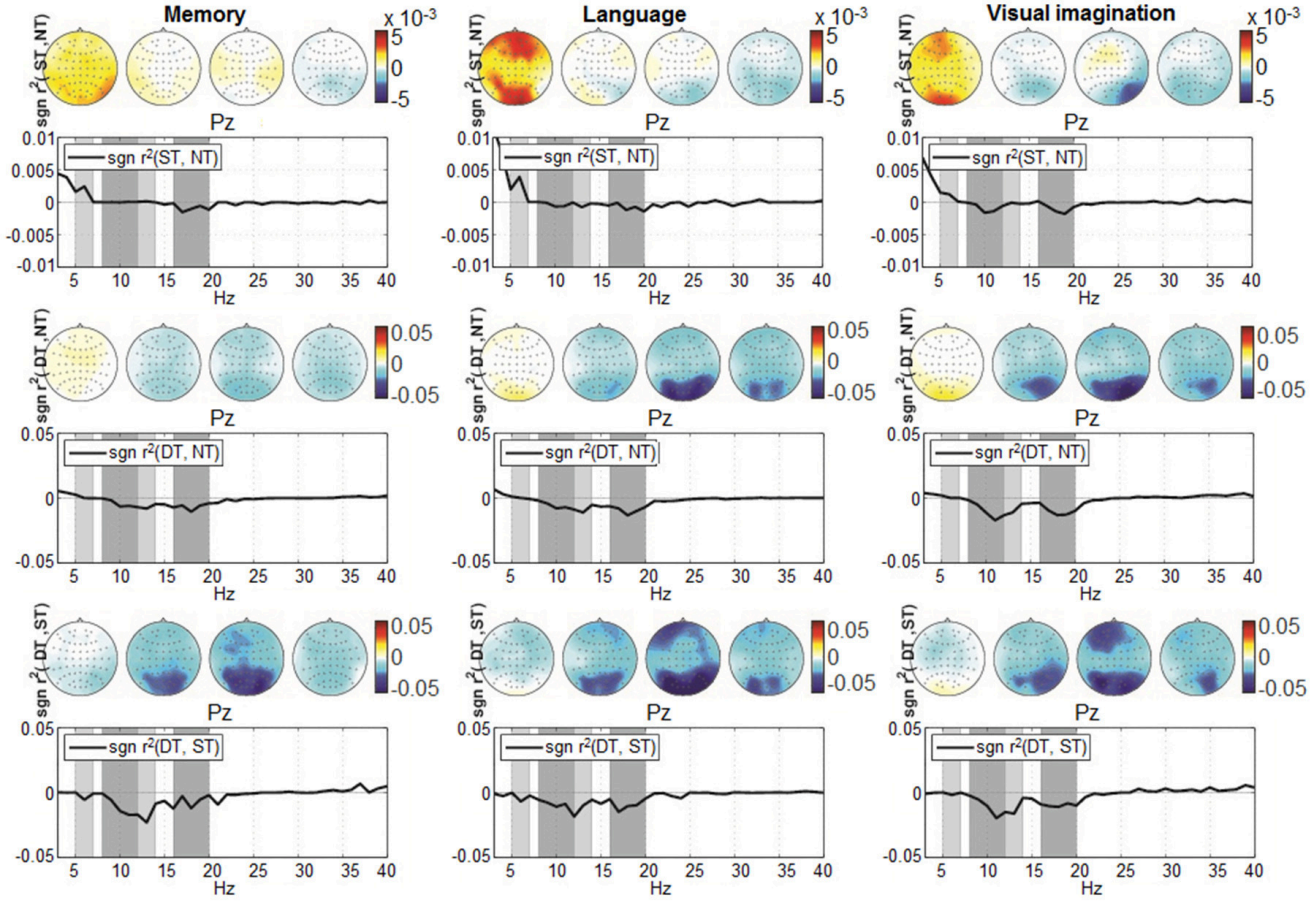
Grand-average spectrum discrimination given by *sgn r*^2^ at location Pz, computed over the entire trial timing (0–2,000 ms) in the frequency range from 3 to 40 Hz. The discrimination pairs ST-NT, DT-NT, and DT-ST are represented from top to bottom, while from left to right are represented according to the three *conditions*: *memory, language*, and *visual imagination*. The four scalp plots refer to discriminative signed *r*^2^-values, corresponding to θ (5–7 Hz), α (8–12 Hz; 12–14 Hz), β (16–20 Hz) frequency bands (shaded in gray). Note the upper graphs scale −0.01 to 0.01, compared to the other scales −0.05 to 0.05.

For the distinction between shallow and deep processing (Figure 7, the bottom graphs), we investigated the average spectrum (mean over trials and participants) given by the signed *r*^2^ over the parietal site (Pz) in the frequency range from 3 to 40 Hz. We observed a higher discriminative difference in the α band (8–14 Hz) and smaller in the β band (16–20 Hz). Due to their prominent difference, also in the scalp maps at frontal and parietal sites, both frequency bands were selected for the analysis in a multi-band approach. A modulation appears also in the θ band (5–7 Hz), visible when comparing shallow with no-processing and deep with shallow processing (upper and bottom graphs). This effect was less marked compared to the other frequency bands and it was not encountered in all levels of processing, therefore it was not considered for the analysis.

**Figure 8:**
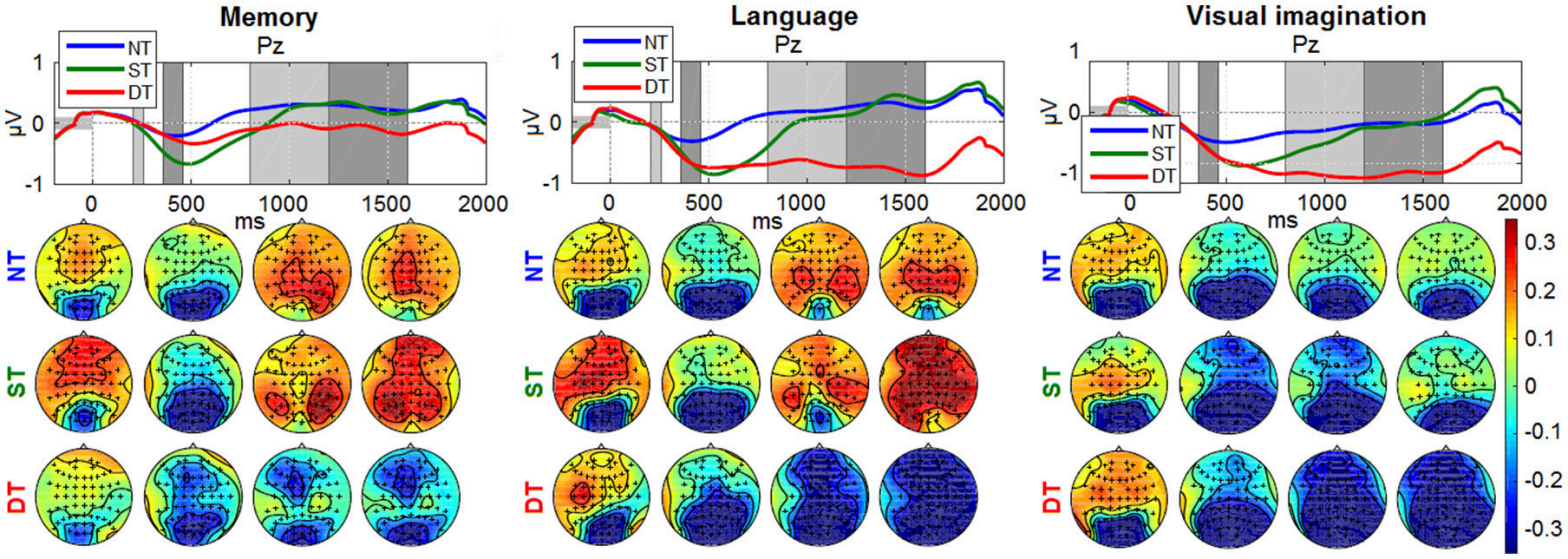
Grand-average ERDs on 8–14 Hz for all processing levels (NT, ST, DT) at electrode Pz. The baseline interval is represented with a gray horizontal bar, equivalent to 200 ms of pre-stimulus interval. The amplitude range was chosen the same for all graphs, as −1 to 1 μV for the time evolution and −0.35 to 0.35 μV for the scalp distributions. The timing intervals for the scalp plots are: 210–260, 360–460, 800–1,200, 1,200–1,600 ms.

**Figure 9:**
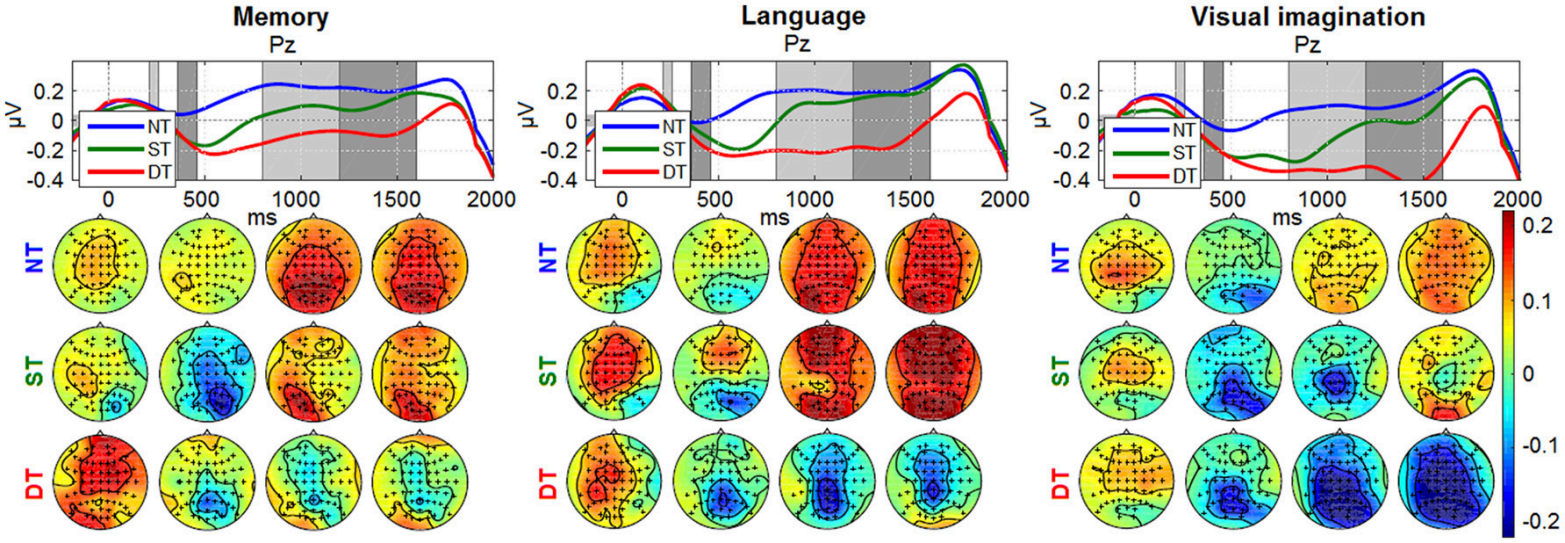
Grand-average ERDs on 16–20 Hz at electrode Pz for all processing levels (NT, ST, DT) with 200 ms baseline. Same amplitude range for all *conditions*, as −0.4 to 0.4 μV for time evolution and −0.22 to 0.22 μV for scalp distributions.

Figures 8, 9 depict the grand average desynchronization and synchronization effects in the 8–14 Hz and 16–20 Hz frequency band which start about 300 ms after stimulus. The time evolution is initially similar for all levels and *conditions* until 300 ms, representing the same amount of evaluating the stimulus information. After this point, the effect of the desynchronization appears, climaxing around 500 ms and it follows a synchronization around 800–1,800 ms. For visualization, we chose the central parietal electrode Pz (for other electrode patterns, see Supplementary Figures S1, S2 in Supplementary Presentation 1). It can be noticed that shallow or deep processes (DT) elicits an attenuation of brain rhythms in comparison to the reference of no-processing (NT). While the ERDs in the ST and DT levels start similarly, they are markedly more sustained in the DT *level*. The effect is more pronounced for the α band as −1 to 0.5 μV and less for the β band, as −0.4 to 0.4 μV. Looking over the scalp distributions, we clearly see higher synchronization (ERS) for the shallow processing (0.1–0.35 μV) compared to the reference no-processing and more pronounced desynchronization (ERD) for a more complex processing (−0.3–0.1 μV). Comparing between processes, a higher ERS in amplitude and spatial distribution is encountered for the *memory* process, contrasting to a more pronounced ERD in the *language* and *visual imagination* case.

Next, the CSP analysis provides a deeper view of the neural oscillations and activity related to cognitive processes. The patterns provide information about the presumed sources of the neural activity which are then optimally projected on the surface of the scalp.

Considering the selected frequency bands, the CSP for participant P4 are shown in the Supplementary Presentation 1 as scalp topographies, computed for all pairs of class combinations (Supplementary Figures S3, S4).

### Classification

The evaluation of the binary multivariate classification based on the combined spatio-temporal (ERP) and multi-band CSP (mCSP with SSD) features, are presented hereunder. The results of classification based on ERP only were presented in Nicolae et al. (2015b).

**Figure 10:**
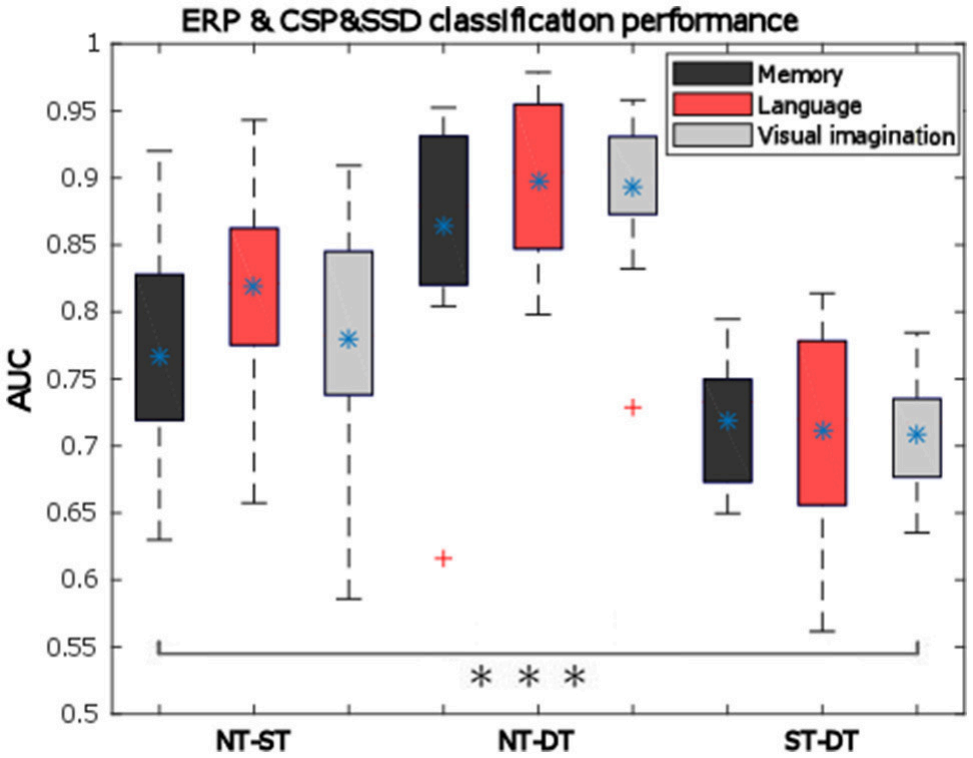
Pairwise classification mean performance over all trials for all cognitive processes (memory—dark gray, language—light red, visual imagination—light gray) given by the area under the ROC curve (AUC) based on ERP-mCSP. The bottom and upper whiskers of each box-plot corresponds to the minimum and maximum values regarding all participants, the rectangular horizontal sides of the box represent the 25 and 75% percentiles of the data, the blue asterisk (^*^) represents the mean values, and red crosses indicates outliers. All pairs show statistically significant AUC, marked with three black asterisks (^***^) in the bar plot (*p* < 0.001).

The general classification performance across participants given by the area under the ROC curve is presented as box-plots in Figure 10. Here we observe good performance which are on average above 70% for ST-DT discrimination, around 75–80% for NT-ST pair and the highest performance for NT-DT discrimination, around 85–90%. All performances for all participants are significantly above chance level (indicated by *t*-test with alpha = 0.01). A two-way repeated measures ANOVA was performed over the AUC values with the factors: *condition* and classification pairs, which provide a statistically significant difference between the classification pairs (*p* < 0.001, *F* = 64.99). Based on the *condition* factor, the results in Figure 10 expose the highest average AUC for the *language condition*, but this observation was not statistically significant (*p* = 0.2112). The distribution of the data was verified using the one-sample Kolmogorov–Smirnov test, supposing the null hypothesis of standard normal distribution samples. The null hypothesis was rejected below the 1% significance level.

With standardization of the feature vectors, the performance is actually increased by 1–3%, compared to the results in Figure 10 with no normalization, although this effect is not statistically significant (*n*-way ANOVA: *p* = 0.0601).

Now relating the classification results to the signed *r*^2^ discrimination for the ERD/ERS curves, it is important to notice that even when no substantial difference was encountered for the NT-ST pair, the combined classification still performed considerably, which was due to the ensemble features approach that integrated also the temporal features. Moreover, when discriminating the deep processing, the classification is significantly improved in the presented combined approach compared to the separate temporal classification results (Nicolae et al., 2015b): increasing from 83 to 87% on average for the NT-DT pair (*p* = 0.0027; *F* = 9.57) and from 68 to about 72% for the ST-DT pair (*p* = 0.0234; *F* = 5.33). No statistical significant difference was obtained for the NT-ST pair. Additionally, while considering only the spectral features, using the SSD method before CSP boosts the classification performance significantly for all cases: for example, from 61 to 74% on average for the ST-DT pair considering the *visual imagination condition* (statistically significant: *p* = 0.0001 with the two-way ANOVA test).

## Discussion and conclusion

We investigated the neural correlates of cognitive processing that indicate the level of profoundness. Effects have been found in ERPs as well as in brain rhythms.

In the ERPs, two peaks arise: one at 250 ms and one prolonged at 400 ms. The first peak refers to the first decision on the type of the stimulus (NT, ST, or DT), based on appearance (color and category) which is similar between different levels of processing. the second peak relates to no processing (NT), mild (ST), or intense (DT) processing, and is graded in amplitude in relation to the corresponding level of processing. The latencies are similar because the levels of processing involve to the same decision task, mental computation. Further, in the deep processing task another decision is involved, deciding the task fulfillment or not, and hence different latencies will be observed trial to trial, which are not clearly visible on grand averages.

The power spectrum analysis, over a wide frequency range from 3 to 40 Hz, provided an overview of the spectral components which were pronounced for the α (8–14 Hz) and the β band (16–20 Hz) and reduced for the θ band (5–7 Hz). Analyzing the components specific to each frequency band, we observed the present activity in the θ band being more pronounced for the *memory* and *language* condition, which reflects the *memory* retrieval activity (Meyer et al., 2015), cognitive processes (Klimesch, 1999), and sustained attention (Huang et al., 2007). However, the signed *r*^2^ differences occurred in the θ band (see Figure 7) are substantially smaller than the differences occurred in the α and the β band. Moreover, when testing the classification considering the θ band only, the performances were around chance level for all discrimination pairs, as expected (e.g., 0.51, 0.56, 0.51 mean AUCs for the *language* condition) which do not show significance with *t*-test at alpha = 0.0056 with Bonferroni correction for multiple comparisons, given *p* > 0.0195 for the NT-ST and ST-DT classification pairs and show barely significance for the NT-DT discrimination with *p* = 0.0052. Therefore, the decision was made to discard this band from further analysis, since in our case it did not produce significant differences. The ERD/ERS curves of the remaining frequency bands display a marked ERD with a peak at a latency of about 500 ms. The duration of the ERD is modulated by the degree of cognitive processing. In addition, strong discrimination in the α band elucidates mental coordination (Palva and Palva, 2007), alert states (Klimesch, 1999), cognitive processing, access to stored information (Klimesch, 2012), and is completed with strong β band differentiation that corresponds to complex mental process and analyzing the presented information (Lachaux et al., 2005).

Comparing across the *conditions*, the activity for *memory* is focused in the temporal and frontal area. More lateralized activity is observed for *language* and more accentuated in parietal and temporal area for *visual imagination* depicting *memory* access and interpretation (Ganis et al., 2004). Comparing the ERD/ERS effects between conditions, a more increased synchronization (higher DT in amplitude and power) is visible for the *memory condition* in comparison with the others, representing an easier process. This effect, contrasts with the behavioral point of view, where more participants stated, on average, the *language* as the easiest method (difficulty scores in Figure 5), effect found statistically significant with *p* = 0.0451 given by a one-way ANOVA statistical test considering one factor (subjects) and three levels (conditions).

In order to improve the oscillatory discrimination, CSP filtering was employed to obtain spatial filters that distinguish the areas where the activity is differentiated between two processing levels (Supplementary Figures S3, S4 in the Supplementary Presentation 1).

For classification, we employed an approach which combines spatio-temporal features with spatio-spectral features. The average AUC is over 76% for the NT-ST pair, more than 86% for the NT-DT pair and 70% for the ST-DT pair. Note, that classification was challenged by the fact that participants moved their eyes freely between the two objects of each stimulus. In addition, it seems probably that the eye movements might have different dynamics between the tasks (e.g., DT might induce more alternations of the gaze between the two objects of a stimulus). For that reason, appropriate artifact removal strategies (ICA with MARA) and careful verification was performed. The resulting ERD and CSP patterns (cf. Figures 8, 9 and Supplementary Figures S3, S4 in Supplementary Presentation 1) do not suggest influences from eye movements, like strong lateralized activity in the frontal area which would be expected to result from horizontal movements. This gives a strong indication that the classification performance was not based on eye movement dynamics but on neural correlates of the cognitive processing. Furthermore, the evaluation of potential contamination by artifacts becomes more robust when analyzing the level of decoding based on the EOG activity alone. Specifically, the classification was performed considering two feature channels (EOG and Fp1; F10 and F9 channels difference) corresponding to the vertical and horizontal eye movements. The results show significant values at chance level for all conditions and classification pairs (e.g., mean AUCs for the *language* condition considering the classification pairs: 0.51, 0.55, 0.46) with *p* > 0.0198 and Bonferroni corrected for the nine comparisons with alpha = 0.0056.

In our experimental paradigm, different cognitive levels were externally imposed using task instructions. The graduated differences observed in the ERPs between shallow and deep processing correlate with different levels of processing, evidenced also in the ERDs/ERSs and are not generated by the targets occurrence, namely the odd-ball effect, which was controlled by imposing the same percentage of the stimuli between ST and DT (12.5%). When comparing the shallow and deep processing with no processing, it cannot be disentangled which differences in the ERPs are generated by the rarity of the occurrence (oddball effect) and which by the additional processing demands of the task. In particular for the ERDs in Figure 8 the time course (differences extending far past 500 ms) seems to suggest that the main difference is due to the additional cognitive processing in ST and DT. This design was chosen in order to have better control of the true level of cognitive processing. For the application perspective, however, we strive to estimate the momentary level of cognitive processing within the natural fluctuations.

In conclusion, the performed investigation of the depth of cognitive processing brings us closer to real scenarios. Compared to standard BCI research our study induced different levels of cognitive processing by tasks that go beyond a simple target/non-target discrimination. Moreover, the visual stimuli used had a higher variability, including the need of eye movements in the exploration of the complex stimuli, which were composed of two objects side-by-side. Our work extends also previous investigation of the effect of task complexity on ERPs and brain oscillations. Again, the set of stimuli used in our study is richer and more complex and importantly, methods from machine learning have been employed for single-trial classification, in a binary approach.

Overall, the present study is a step forward toward applications that estimate the level of cognitive processing in realistic settings of human-computer interaction (Gamberini et al., 2015) and in safety critical workplaces (Venthur et al., 2010). A further step in order to discriminate the ongoing level of cognitive processing is to apply a regression approach, as in Naumann et al. (2017). A different perspective for the current study is the development of techniques suitable for adaptive learning environments based on user state monitoring regarding the depth of cognitive processing. In this regard, it would be interesting to augment the approach with predicting remembered vs. not remembered items (Klimesch et al., 1996) in *memory* tasks.

## Data availability statement

The EEG signals and behavioral data acquired in this study will be made available from the DepositOnce repository of Technische Universität Berlin (https://depositonce.tu-berlin.de/).

## Author contributions

Design of the study by BB. Implementation and data acquisition by IN. IN performed data analysis and LA and BB reviewed and revised the analysis. IN wrote the manuscript draft which was reviewed and revised by LA and BB.

### Conflict of interest statement

The authors declare that the research was conducted in the absence of any commercial or financial relationships that could be construed as a potential conflict of interest.

## Supplementary material

Supplementary Video 1 Short example with 30 trials for the experimental paradigm considering the *memory* condition. First, the visual questionnaire is presented evaluating participant's current mental state (good, ok, bad). Next, thetarget pair (pear, banana) specifying the target category (fruits) and the color target (red) is presented along with the current condition that needs to be performed(*memory*). When the participant memorized the target, he/she press a keyboard button and the sequence starts after a short countdown. At the end of the run, the participant enters the number counted and receives feedback considering the correct number. In total, there were 4 DT (from which two fulfilled the *memory* question task), 7 ST, and 19 NT stimuli presented, accumulating a final number counted of 31. Note that the number of trials is higher in the actual run of the experiment (120 stimuli) and the ratio of the stimuli is also different (75:12.5:12.5 ± 2% for NT:ST:DT), while this example may seem harder (with a ratio of 63.3:23.3:13.3 (%) for NT:ST:DT).

Supplementary Video 2 Short example with 30 trials for the experimental paradigm considering the *language* condition. The target pair here (submarine, submarine) refer to the mobility category and shows the color target (green). The current condition that needs to be performed is also specified (*language*). In total, 4 DT (from which two fulfilled the *language* question task), 7 ST, and 19 NT stimuli were presented, resulting in a final number counted of 31. Same notice as in the memory example, that the number of trials and the ratio are different here compared with the actual experiment.

Supplementary Video 3 Short example with 30 trials for the experimental paradigm considering the *visual imagination* condition. The target pair here (cow, elephant) and the color target (red) are presented along with the specification of the current condition that needs to be performed (*visual imagination*). In total, 4 DT (from which two fulfilled the *visual imagination* question task), 5 ST, and 21 NT stimuli were presented, resulting in a final number counted of 29. Same notice as in the memory and language example, that the number of trials and the ratio (70:16.67:13.3 (%) for NT:ST:DT) are different compared with the actual experiment.
