# Supplementary material for: Assessing the Depth of Cognitive Processing as the Basis for Potential User-State Adaptation
Source: Front Neurosci. 2017 Oct 4;11:548. doi: 10.3389/fnins.2017.00548 (PMC5632679; doi:10.3389/fnins.2017.00548)
Supplement: Supplementary file 4 [file Presentation1.pdf]

Supplementary Material

Assessing the depth of cognitive processing for user-state adaptation

Irina-Emilia Nicolae*, Laura Acqualagna, Benjamin Blankertz*

*** Correspondence:** Irina-Emilia Nicolae: irina.nicolae@aut.pub.ro
Benjamin Blankertz: benjamin.blankertz@tu-berlin.de

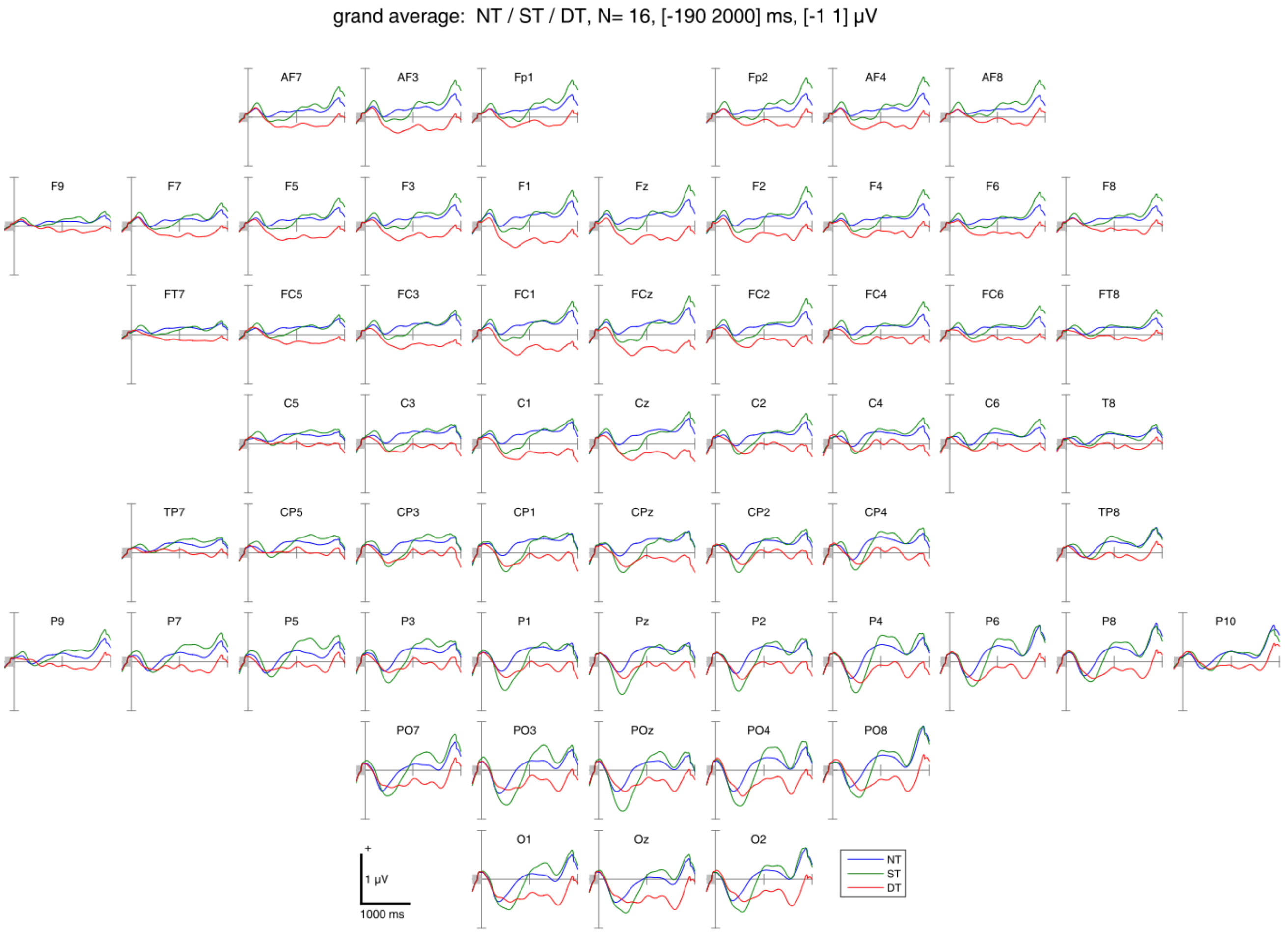

Supplementary Figure S1. Grand average ERD/ERS curves on 8-14 Hz considering all electrodes and all processing levels for the memory *condition*

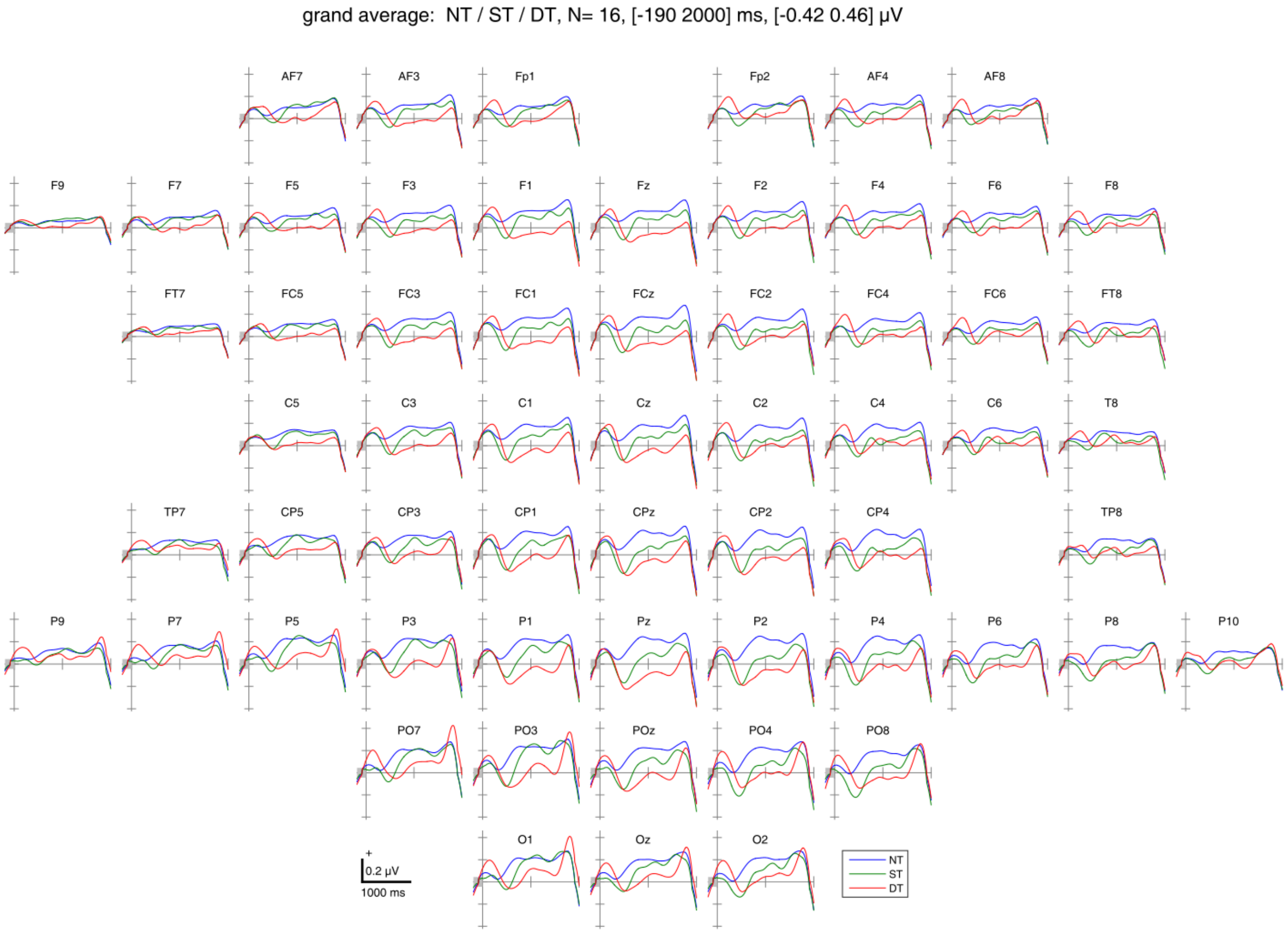

Supplementary Figure S2. Grand average ERD/ERS curves on 16-20Hz considering all electrodes and all processing levels for the memory *condition*

The Supplementary Figure S3 shows the patterns for the α frequency band (8-14 Hz) and the patterns of the β band (16-20 Hz) are presented in Figure S4. Participant P4 shows clear patterns, more pronounced for 8-14 Hz compared to 16-20 Hz. The first components refer to a maximization of the variance for the first class and a minimization of the variance for the second class (largest eigenvalue). The last components represent the second class maximization of the variance and in the same time first class minimization of the variance (smallest eigenvalue). Here, it is important to notice that the interpretation focuses only on the spatial patterns, without considering the spatial filters which cannot be interpretable (Haufe *et al.*, 2014; Bießmann *et al.* 2012). Participant P4 chosen for visualization showed the highest classification accuracy on average (77% accuracy for ST-DT, 92% for NT-DT and 80% for NT-ST) and the best behavioral response (46.66% perfect runs out of 15, with an average behavioral ratio considering all conditions of 0.04708). The neural correlates of no-processing show diffused activation patterns at different scalp locations in comparison with the cognitive processes, where the shallow and deep processes illustrate localized activity in the central or parietal area, with increased or decreased variance (green or purple).

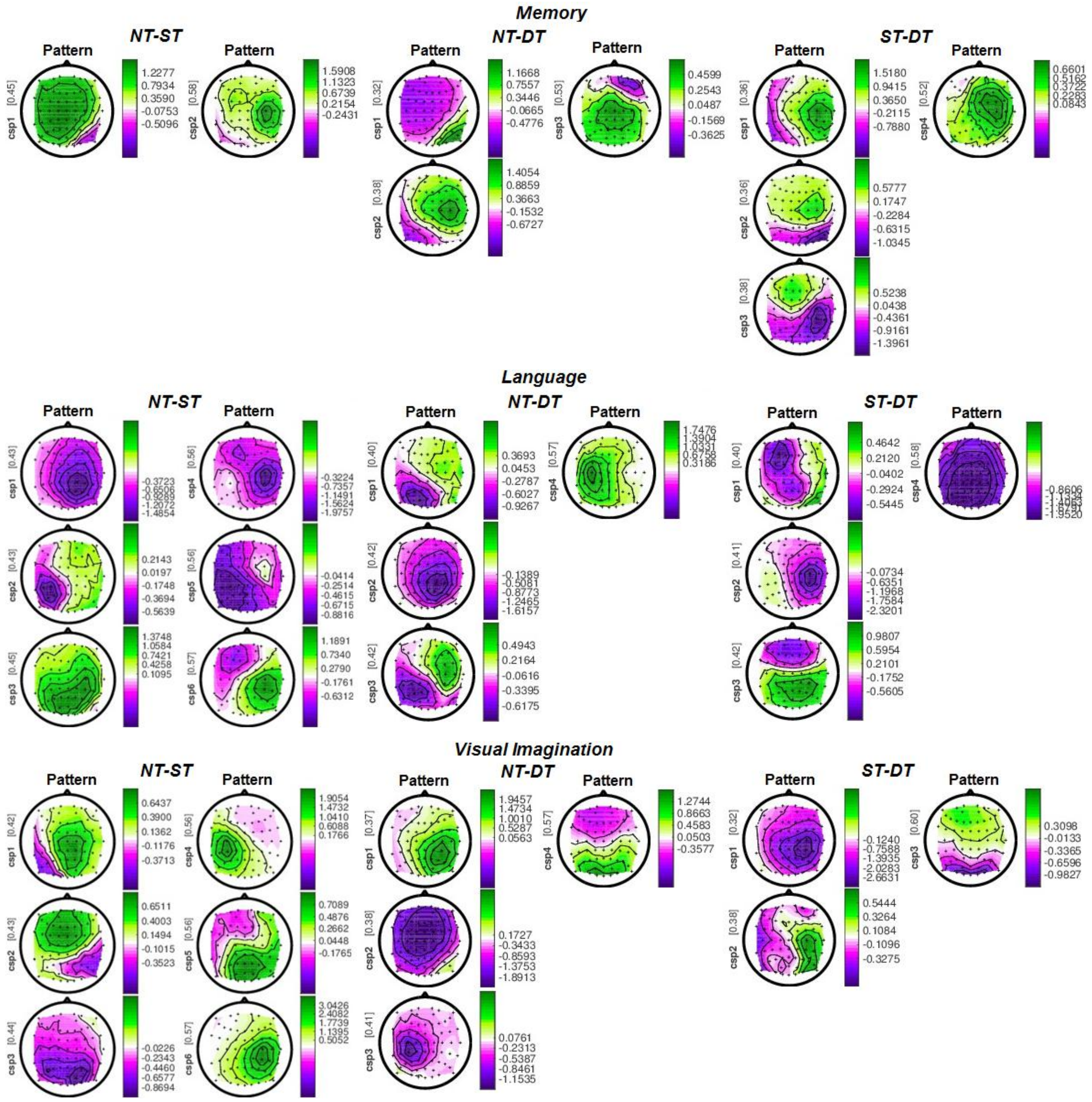

Supplementary Figure S3. CSP analysis (patterns) with preliminary SSD related to the 8-14 Hz frequency band considering NT-ST, NT-DT, ST-DT class pairs for each *condition* (Participant P4). The components with value less than 0.5 best represents the maximization of the first class and the others greater than 0.5 represent the maximization of the second class. Because the CSPs are computed for each fold, inside the cross-validation, we present here the patterns corresponding to the first fold of cross-validation. In order to visualize this spatial representation over the entire scalp map, the CSP filters are projected back to the original space.

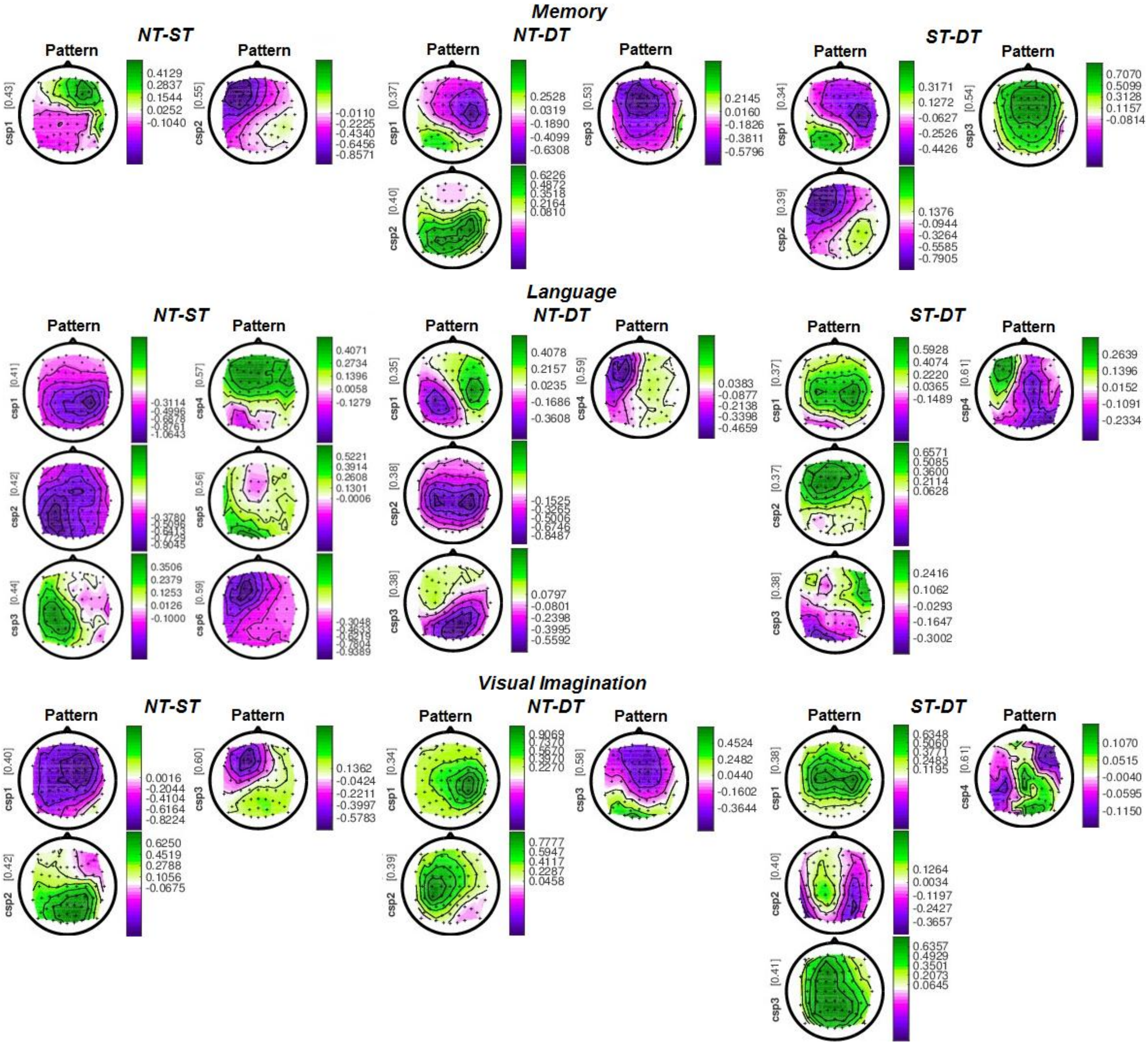

Supplementary Figure S4. CSP patterns for the 16-20 Hz frequency band for all *conditions* and classification pairs (Participant P4). CSPs for the first fold of cross-validation.

References

Bießmann, F., Dähne, S., Meinecke, F. C., Blankertz, B., Görgen, K., Haufe, S. (2012). „On the interpretability of linear multivariate neuroimaging analyses: filters, patterns and their relationship." In *Proc. of the 2nd NIPS Workshop on Machine Learning and Interpretation in Neuroimaging*, Lake Tahoe, Nevada, United States.

Haufe, S., Meinecke, F., Görgen, K., Dähne, S., Haynes, J., Blankertz, B., et al. (2014). On the interpretation of weight vectors of linear models in multivariate neuroimaging. *Neuroimage.* **87**: 96-110. doi: 10.1016/j.neuroimage.2013.10.067
